# Percutaneous biopsy of abdominal lesions: what is currently the best diagnostic strategy?

**DOI:** 10.1590/0100-3984.2018.51.3e1

**Published:** 2018

**Authors:** Thiago Franchi Nunes

**Affiliations:** 1 MD, PhD, Interventional Radiologist and Angiographer, Head of the Department of Interventional Radiology of the Hospital Universitário Maria Aparecida Pedrossian da Universidade Federal de Mato Grosso do Sul (HUMAP-UFMS), Campo Grande, MS, Brazil. E-mail: intervencao.radiologia@gmail.com.

Surgical or imaging-guided percutaneous biopsy is an extremely important part of the diagnosis, staging, and follow-up of suspected or known malignancies. In the past, surgical biopsy procedures were necessary in order to obtain sufficient samples of the lesion for the appropriate pathological diagnosis. However, in addition to being invasive, surgical biopsies are associated with high morbidity rates^(^^1^^)^.

Imaging-guided percutaneous biopsy procedures have been increasingly used, largely because of their noninvasive nature, low complication rates, and lower cost in comparison with surgical methods^(^^2^^)^. The coaxial technique, using a larger external guide needle, offers additional advantages, especially for deep or difficult-to-access lesions^(^^3^^-^^5^^)^. This technique offers greater precision, increasing the quantity of fragments collected for pathological analysis, reducing the risks of complications and, especially, of tumor dissemination along the needle path.

Ultrasound-guided percutaneous procedures have numerous advantages for the diagnosis of abdominal lesions^(^^6^^,^^7^^)^: wide availability and accessibility of the method; absence of ionizing radiation; short procedure time; real-time visualization of the biopsy needle and the target lesion during the procedure; ability to guide the procedure in almost any anatomical plane; and low cost. However, the success of ultrasound-guided percutaneous biopsy depends on a number of factors, including the experience and advanced knowledge of the technique on the part of the interventional radiologist. Ultrasound also has advantages in guiding biopsies with intracavitary access, which are traditionally used in cases of prostate biopsy. They are also considered excellent alternatives for the diagnosis of adnexal/parametrial, uterine, and low rectal lesions, such as stromal tumors and presacral lesions. Some software for the fusion of ultrasound images and images obtained with magnetic resonance imaging has shown promise^(^^8^^)^.

Computed tomography (CT)-guided puncture, which has been one of the most widely used techniques in interventional radiology, includes biopsies, drainage, and radiofrequency ablation procedures^(^^1^^,^^2^^,^^9^^)^. We believe that CT is superior to the ultrasound for accessing retroperitoneal lesions, mesenteric lymph nodes, and some deep pelvic lesions, as well as target-organ lesions not visualized by ultrasound. CT-guided percutaneous biopsies of omental and mesenteric lesions have high rates of technical success and diagnostic yield, regardless of lesion size or skin depth^(^^10^^)^. Some authors have also demonstrated the benefit of techniques guided by PET/CT scans^(^^11^^)^, in comparison with conventional CT, for cases in which the lesions have extensive necrotic content, with the object of directing the material collection to the regions with high FDG uptake; however, the disadvantages of that technique, such as cost and logistics, often make it unfeasible.

Like ultrasound-guided biopsies, CT-guided biopsies have some negative aspects. The conventional puncture technique has low real-time guidance capability to track the needle and the target location. A step-by-step, intermittent sweep of the region of interest is necessary to confirm the location of the needle each time it is advanced, thus increasing the procedure time. At centers that are more modern, where CT fluoroscopy equipment is available, guided punctures become even faster and more precise. However, for both techniques (conventional CT and CT fluoroscopy), patients are exposed to high radiation levels.

The article “Computed tomography-guided percutaneous biopsy of abdominal lesions: indications, techniques, results, and complications”^(^^12^^)^, published in this issue of Radiologia Brasileira, recounts an excellent study in which the authors evaluated the performance of CT-guided percutaneous biopsy of abdominal lesions, demonstrating excellent performance of the technique, at an interventional radiology teaching referral center for cancer. The authors showed that the technique has good diagnostic accuracy and low complication rates, corroborating other results described in the literature.

Some other biopsy techniques are being used with ever-increasing frequency, such as those involving luminal access. They are ultrasound- and fluoroscopy-guided techniques using forceps and introducer sheaths. The main indications for the use of such techniques are bile duct tumors (Klatskin tumors), urothelial (kidney and ureter) lesions, and intravascular lesions^(^^13^^-^^15^^)^. In the case of renal and biliary tumors, these techniques provide, in addition to biopsy of the tumor lesion, the possibility of placement of external drains in the same surgical procedure. Because patients usually present with dilatation of the bile ducts, necessitating biliary drainage in cases of hepatic tumor (cholangiocarcinoma) or hydronephrosis with post-renal insufficiency (in some cases of urothelial tumors), requiring percutaneous nephrostomy.

Conceptually, interventional radiology procedures provide less risk, less pain, and shorter recovery times than do surgical and other conventional procedures. We believe that the tendency would be to choose the biopsy procedure technique and the imaging method, be it ultrasound, CT, fluoroscopy, a combination of those, or even a more modern method such as PET/CT, on a case-by-case basis. In all cases, a multidisciplinary approach should be employed and the procedures should be performed by specialists and properly certified professionals.
